# A pentaplex PCR assay for the rapid detection of methicillin-resistant *Staphylococcus aureus *and Panton-Valentine Leucocidin

**DOI:** 10.1186/1471-2180-9-113

**Published:** 2009-05-28

**Authors:** Hassanain Al-Talib, Chan Yean Yean, Alyaa Al-Khateeb, Habsah Hassan, Kirnpal-Kaur Banga Singh, Karim Al-Jashamy, Manickam Ravichandran

**Affiliations:** 1 Department of Medical Microbiology and Parasitology, School of Medical Sciences, Universiti Sains Malaysia Kota Bharu, Malaysia; 2 Human Genome Center, School of Medical Sciences, Universiti Sains Malaysia, Kota Bharu, Malaysia; 3 Faculty of Health & Life Sciences, Management & Science University, Shah Alam, Malaysia; 4 Faculty of Applied Sciences, AIMST University, Kedah, Malaysia

## Abstract

**Background:**

*Staphylococcus aureus *is a major human pathogen, especially methicillin-resistant *S. aureus *(MRSA), which causes a wide range of hospital and community-acquired infections worldwide. Conventional testing for detection of MRSA takes 2–5 days to yield complete information of the organism and its antibiotic sensitivity pattern.

**Results:**

The present study focused on the development of a pentaplex PCR assay for the rapid detection of MRSA. The assay simultaneously detected five genes, namely 16S rRNA of the *Staphylococcus *genus, *fem*A of *S. aureus*, *mec*A that encodes methicillin resistance, *luk*S that encodes production of Panton-Valentine leukocidin (PVL), a necrotizing cytotoxin, and one internal control. Specific primer pairs were successfully designed and simultaneously amplified the targeted genes. The analytical sensitivity and specificity of the pentaplex PCR assay was evaluated by comparing it with the conventional method. The analytical sensitivity of the pentaplex PCR at the DNA level was found to be 10 ng DNA. The analytical specificity was evaluated with 34 reference staphylococci and non-staphylococcal strains and was found to be 100%. The diagnostic evaluation of MRSA carried out using 230 clinical isolates, showed 97.6% of sensitivity, 99.3% of specificity, 98.8% of positive predictive value and 98.6% of negative predictive value compared to the conventional method. The presence of an internal control in the pentaplex PCR assay is important to exclude false-negative cases.

**Conclusion:**

The pentaplex PCR assay developed was rapid and gave results within 4 h, which is essential for the identification of *Staphylococcus *spp., virulence and their resistance to methicillin. Our PCR assay may be used as an effective surveillance tool to survey the prevalence of MRSA and PVL-producing strains in hospitals and the community.

## Background

*Staphylococcus aureus *is a facultative pathogenic Gram-positive bacterium that is well known as colonizer of the human skin, and is a leading cause of diseases ranging from mild skin and soft tissue infections to life-threatening illnesses, such as deep post-surgical infections, septicemia and toxic shock syndrome [1]. Methicillin-resistant *S. aureus *(MRSA) and methicillin-sensitive *S. aureus *(MSSA) are responsible for a large proportion of nosocomial infections, which makes treatment difficult [2]. During the past decade, an increasing number of MRSA cases has been encountered globally among healthy community residents [3]. These isolates are referred to as community-acquired MRSA (CA-MRSA), which are genetically and phenotypically different from representative hospital-acquired MRSA (HA-MRSA), in relation to their antibiotic resistance patterns, and by the allocation of their staphylococcal chromosomal cassette (SCC*mec*) types, IV and V [3,4]. Coagulase-negative staphylococci (CoNS) were regarded as harmless skin commensals prior to the 1970s; however, they are now recognized as important causes of human infections [5,6]. CoNS are also among the most commonly isolated bacteria in clinical microbiology laboratories [7]. Furthermore, CoNS often serve as reservoirs of antimicrobial-resistance determinants, since they usually have a high prevalence of multidrug resistance. Therefore, it is important to describe and distinguish *S*. *aureus *strains and CoNS [8]. Methicillin resistance in staphylococci is mainly mediated by the over-production of PBP2a, an additional modified penicillin-binding protein with low affinity for β-lactam antibiotics. The *mecA *gene, the structural determinant that encodes PBP2a, is therefore considered as a useful molecular marker of putative methicillin resistance in *S*. *aureus *and CoNS [9,10].

Clinical laboratory tests for methicillin resistance are highly dependent on growing conditions such as temperature, pH and salt concentration [11]. Thus, these factors emphasize the need to develop a rapid, accurate and sensitive method for detection of methicillin-resistant staphylococci, which does not depend on growth conditions. Nucleic-acid-based tests using PCR are increasingly being used in laboratories to replace time-consuming, labor intensive and less sensitive conventional diagnostic methods, such as biochemical identification and Kirby-Bauer antimicrobial susceptibility tests. Various PCR methods have been developed to identify: (i) *Staphylococcus *genus [12]; (ii) methicillin-resistance [13]; and (iii) Panton-Valentine leukocidin (PVL)-producing *Staphylococcus *genus [14]. These methods do not detect all of the above-mentioned targets simultaneously. Hence, the present study focused on the design of a pentaplex PCR for methicillin-resistant staphylococci with an internal control for the detection of *Staphylococcus *genus (16S rRNA gene), methicillin-resistant staphylococci (*mec*A gene), community-acquired MRSA (*luk*S gene), and discrimination between *S. aureus *and CoNS (*fem*A gene).

## Results

**Figure 1:**
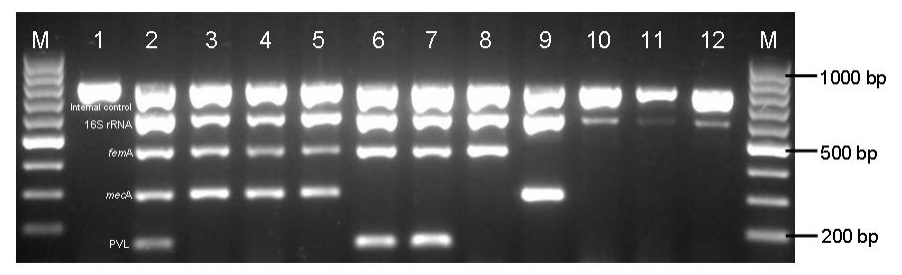
**Pentaplex PCR assay profile with reference strains**. M, 100-bp marker; lane 1, negative control; lane 2, Staphylococcal positive control; lane 3, ATCC 33591 (16S rRNA, *fem*A-*S. aureus, mec*A); lane 4, ATCC 33592 (16S rRNA, *fem*A-*S. aureus, mec*A); lane 5, ATCC 43300 (16S rRNA, *fem*A-*S. aureus, mec*A); lane 6, ATCC 25923 (16S rRNA, *fem*A-*S. aureus, luk*S); lane 7, ATCC 49775 (16S rRNA, *fem*A-*S. aureus, luk*S); lane 8, ATCC 51153 (16S rRNA, *fem*A-*S. aureus*); lane 9, CoNS methicillin-resistant clinical isolate (16S rRNA, *mec*A); lane 10, ATCC 14990 (16S rRNA); lane 11, ATCC 29970 (16S rRNA); lane 12, ATCC 13518 (16S rRNA); M, 100-bp marker

In the present study, the pentaplex PCR was optimized successfully to identify the *Staphylococcus *genus (16S rRNA), *S. aureus *species (*fem*A), methicillin resistance (*mec*A) and PVL toxin (*luk*S) genes simultaneously. Stepwise optimization of primer concentration, annealing temperature, MgCl_2_, dNTP and Taq polymerase was carried out. The pentaplex PCR gave the best results when 3.13 mM MgCl_2_, 200 μM dNTP, 0.75 U Taq polymerase and 60°C annealing temperature were used. The analytical sensitivity of the pentaplex PCR at the DNA level was found to be 10 ng DNA (data not shown), whereas, at the bacterial level, it was found to be 10^4 ^CFU/mL (data not shown). The analytical specificity of the pentaplex PCR assay at the genus level was determined using 10 staphylococcal reference strains and found to be positive for the *Staphylococcus *genus specific 16S rRNA gene. A representative gel picture of methicillin resistance with reference strains is shown in Figure 1, while the other 10 Gram-positive non-staphylococcal and 13 Gram-negative strains were negative. All the reference strains of *S. aureus *were positive for *fem*A gene by pentaplex PCR, while other CoNS species were negative (Table 1). Hence, all methicillin-resistant reference strains were positive for *mec*A gene by pentaplex PCR. However, the methicillin-sensitive reference strains were negative for *mec*A gene by pentaplex PCR (Table 1). Overall, the analytical specificity of pentaplex PCR was 100% for the detection of MRSA reference strains.

**Table 1:** Bacterial species and strains used in this study and results of pentaplex PCR.

No. Reference strains	16S rRNA^a^	*fem*A	*mec*A^b^	*luk*S	**Internal control**
1. *S. aureus *(ATCC 33591)	+	+	+	-	+
2. *S. aureus *(ATCC 33592)	+	+	+	-	+
3. *S. aureus *(ATCC 43300)	+	+	+	-	+
4. *S. aureus *(ATCC 25923)^d^	+	+	-	+	+
5. *S. aureus *(ATCC 49775)	+	+	-	+	+
*6. S. aureus *(ATCC 51153)^e^	+	+	-	-	+
7. *S. epidermidis *(ATCC 14990)	+	-	-	-	+
8. *Staphylococcus haemolyticus *(ATCC 29970)	+	-	-	-	+
9. *Staphylococcus saprophyticus *(ATCC 13518)^d^	+	-	-	-	+
10. CoNS methicillin-resistant^e^	+	-	+	-	+
11. *Streptococcus *spp. Group A (ATCC 19615)^e^	-	-	-	-	+
12. *Streptococcus *spp. Group B (ATCC 12401)^e^	-	-	-	-	+
13. *Streptococcus *spp. Group G^e^	-	-	-	-	+
14.*Streptococcus *spp. Group F^e^	-	-	-	-	+
15. *Bacillus subtilis *(ATCC 6633)^e^	-	-	-	-	+
16.*Listeria monocytogenes *(ATCC 7644)^e^	-	-	-	-	+
17. *Enterococcus faecium *LMG 16192^c^	-	-	-	-	+
18. *Enterococcus faecalis *(ATCC 29212)^e^	-	-	-	-	+
19. *Corynebacterium *spp^e^	-	-	-	-	+
20. *Escherichia coli *(EHEC)^e^	-	-	-	-	+
21. *E. coli *(EPEC)^e^	-	-	-	-	+
22.*E. coli *(ETEC)^e^	-	-	-	-	+
23. *Klebsiella pneumoniae *(ATCC 10031)^e^	-	-	-	-	+
24. *Shigella sonnei *(ATCC 25931)^e^	-	-	-	-	+
25. *Shigella flexneri *(ATCC 12022)^e^	-	-	-	-	+
26. *Shigella boydii *(ATCC 9207)^e^	-	-	-	-	+
27.*Proteus mirabilis *(ATCC 29245)^e^	-	-	-	-	+
28. *Salmonella typhi*^e^	-	-	-	-	+
29. *Pseudomonas aeruginosa *(ATCC 27853)^e^	-	-	-	-	+
30.*Yersinia enterocolitica *(ATCC 23715)^e^	-	-	-	-	+
31. *Vibrio cholerae *(O1 classical)^e^	-	-	-	-	+
32. *Citrobacter freundii *(ATCC 8090)^e^	-	-	-	-	+
33.*Gardnerella *spp^e^	-	-	-	-	+
34.*Candida albicans *(ATCC 10231)^e^	-	-	-	-	+

^a ^*Staphylococcus genus*

^b ^methicillin-resistant genotype

^c ^Reference strains from Belgian Co-ordinated Collections of Micro-organisms (BCCM), Ghent,

Belgium

^d ^Obtained from Institute for Medical Research, Malaysia

^e ^Department of Medical Microbiology and Parasitology, School of Medical Sciences, Universiti

Sains Malaysia.

Upon completion of the standardization of the methicillin-resistant pentaplex PCR assay with reference strains, the assay was validated with 230 clinical isolates. Among these, all had 16S rRNA, 82 contained *mec*A, 178 had *fem*A and none had *luk*S genes by pentaplex PCR. However, by the conventional E-test antimicrobial susceptibility method, 83 of the isolates were methicillin-resistant staphylococci (oxacillin MIC ≥ 8 μg/mL). One of the *S. aureus *isolates that was positive for *mec*A gene by pentaplex PCR was found to be sensitive to oxacillin by the conventional MIC method. The diagnostic accuracy of a pentaplex PCR for 16S rRNA and *fem*A genes was determined using 230 clinical isolates and found to have 100% sensitivity, specificity, and positive and negative predictive values. However, the pentaplex PCR for the *mec*A gene detection showed 97.6% of sensitivity, 99.3% of specificity, and 98.8% of positive and 98.6% of negative predictive values in detecting methicillin-resistant staphylococci.

## Discussion

The present study is believed to be the first to develop a combined molecular test for the rapid identification and discrimination of the *Staphylococcus *genus from others, with simultaneous discrimination of methicillin-resistant from -susceptible staphylococcal strains, *S. aureus *from CoNS, and concomitant detection of PVL genes. Although there are numerous reports on PCR assays for the detection of methicillin resistance [15-17], only a few of them have incorporated internal controls in their assays to rule out false-negative results [18,19]. According to guidelines for Molecular Diagnostic Methods for Infectious Diseases [20], incorporation of an internal control in the reaction is essential for the diagnostic test to exclude false-negative results or the presence of inhibitors [21]. In the present study, the inclusion of a 759-bp internal control in the pentaplex PCR assay helped us to rule out false-negative results or PCR inhibitors. To deal with applicability and accuracy, we further applied our pentaplex PCR assay to test a total of 53 MRSA, 125 MSSA, 22 methicillin-sensitive CoNS, and 30 methicillin-resistant CoNS from routine clinical specimens obtained from Hospital Universiti Sains Malaysia.

The *Staphylococcus *genus consists of at least 35 unique species, and only a few have been recovered from humans [6]. Previously published staphylococcal genus specific primers [22,23] do not target wholly conserved regions in the staphylococcal 16S rRNA gene, which results in misdetection of some important CoNS. Therefore, we designed a new conserved *Staphylococcus *genus-specific primer and included it in our new pentaplex PCR assay, which allowed us to detect most species and strains of staphylococci (Table 1). The pentaplex PCR was found to be 100% sensitive and specific in detecting 16S rRNA genes among staphylococcal strains.

Another gene, *fem*A, has been characterized as essential for the expression of methicillin resistance in *S. aureus *and is universally present only in *S. aureus *isolates. This gene has been implicated in cell wall metabolism and is present in large amounts in actively growing cultures [24]. Specific primers for *fem*A were designed and used in the pentaplex PCR to survey various staphylococcal isolates from our culture collection. All 178 *S. aureus *cultures examined, regardless of the presence or absence of *mec*A, produced a positive result in PCR for *fem*A. In contrast to the results with *S. aureus*, when 52 strains of CoNS were examined for the presence of the *fem*A gene by pentaplex PCR, all were negative. The *fem*A gene in the pentaplex PCR assay was able to rule out non-*S. aureus *staphylococci, as reported by Francois *et al. *[25].

The *mec*A gene is unique to methicillin-resistant staphylococci [26]. The DNA sequences of the *mec*A genes found in *S. aureus *and CoNS are >99% identical [27]. Thus, the *mec*A gene represents a useful molecular component for rapid identification of MRSA and methicillin-resistant CoNS by PCR. One of the 147 MSSA isolates was shown to be *mecA*-positive by pentaplex PCR. Although genotypically the *mec*A gene was detected and confirmed by PCR, it is possible that the *mec*A gene is non-functional (non-PBP-2a producing) and is not expressed phenotypically or due to the presence of pseudogene [28]. Clinically, it is important to differentiate between classical type *mec*A-positive MRSA strains among other borderline-resistant *S. aureus *strains that result from hyperproduction of β-lactamases [11].

The *mec*A-positive isolates were either heterogeneous or homogeneous in their expression of resistance. When heterogeneous isolates are tested by standard conventional methods, some cells appear susceptible and others resistant, while almost all homogeneous isolates express resistance when tested by standard methods [29].

Production of PBP-2a may be stimulated during chemotherapy with β-lactam antibiotics, which converts heterogeneous isolates into oxacillin-resistant strains, therefore, the identification of methicillin-resistant staphylococci in the laboratory is complicated by the heterogeneous nature of the resistance, and by the variables that influence its expression (i.e., medium, inoculum size, pH, temperature, and salt concentration) [30]. For these reasons, detection of *mec*A gene is crucial for precise discrimination of methicillin resistance among staphylococci.

Almost 100% of CA-MRSA strains contain the *luk*S gene, compared to <5% of HA-MRSA. The PVL-encoding gene allows the production of a necrotizing cytotoxin, which may be responsible for staphylococcal invasiveness and virulence [4,31]. We included this gene in the pentaplex PCR assay to categorize our isolates and accurately discriminate CA-MRSA and HA-MRSA.

None of the MRSA, MSSA and CoNS isolates harbor the PVL-encoding *luk*S gene. With regard to MRSA, this is not surprising because all MRSA isolates in our study were nosocomial organisms. A high prevalence of *luk*S gene among MSSA has been reported in the neighboring countries of Singapore and Indonesia, with none and low prevalence of *luk*S gene among MRSA [32,33]. The low prevalence in Malaysia is ascribed to restrictive antibiotic usage and a strict policy of national surveillance for MRSA.

Rapidly increasing prevalence of serious CA-MRSA infections and mortality have been reported globally [34-36], an accurate and rapid method of screening *S. aureus *isolates with *luk*S gene was a vital step for appropriate therapy and controlling the dissemination of this potentially virulent pathogen. In Malaysia, the presence of MRSA has been reported [37,38], and cases of MRSA infection and colonization have also been reported in the neighboring countries of Singapore and Indonesia [32,33].

The presented pentaplex PCR assay is robust and practicable for culture confirmation purposes. However, the 10^4 ^CFU/mL analytical sensitivity of this current pentaplex PCR assay might not sensitive enough for the direct testing of clinical specimens.

A previous study by Gosbell et al, in 2001 confirmed that MRSA-screen test gave excellent sensitivity and specificity for MRSA detection, and was quicker and cheaper than PCR [39], while other study showed lower sensitivity and specificity in detecting methicillin resistance in CoNS [40] and couldn't identify neither PVL toxin encoding gene among staphylococci nor differentiate between CA-MRSA and HA-MRSA. Hence the PCR assay developed in the present study will be useful in the epidemiological screening of MRSA patients or carriers. We are currently evaluating this assay for screening for MRSA carriage in Malaysia.

## Conclusion

The PCR assay was able to detect four genes that are essential for the identification of *S. aureus *and its methicillin-resistant genotypes simultaneously in a single test within 4 h. The built-in internal control in this assay prevented false-negative results. The diagnostic accuracy was determined using 230 clinical specimens and showed 97.6% of sensitivity and 99.3% of specificity in detecting methicillin-resistant staphylococci. Hence, this test can be used as an effective diagnostic and surveillance tool to monitor the spread and emergence of MRSA.

## Methods

### Study design

This was a cross-sectional study in which the retrospective sample size was calculated by using PS software (Dupont & Plummer, 1997) using Dichotomous based on the sensitivity of the E-test and PCR at 100% and 98% respectively [41,42]. The Research and Ethics Committee, School of Medical Sciences, Universiti Sains Malaysia, approved the study protocol.

### Bacterial strains and clinical specimens

The *Staphylococcus *spp. reference strains and other bacteria used in this study are listed in Table 1. A total of 230 retrospective *Staphylococcus *spp. that were isolated from routine clinical specimens obtained from Hospital Universiti Sains Malaysia, from March 2006 to February 2007, were used in this study. Among the 230 clinical isolates, 86 were from nasal samples, 45 from blood samples, 34 from pus samples, 19 each from body fluid, wounds and CSF samples, and eight from urine samples.

### Screening of Staphylococcus spp. from clinical specimens by the conventional method

The clinical isolates were inoculated onto Columbia blood agar (Merck, NJ, USA) plates with 5% sheep blood for 24 h at 37°C. The staphylococcal isolates were identified morphologically and biochemically by standard laboratory procedures [43]. The coagulase plasma test (Remel, Lenexa, KS, USA) was performed on organisms that exhibited typical staphylococcal colony morphology, to allow for discrimination of *S*. *aureus *from CoNS. Susceptibility testing for methicillin resistance and other antibiotic resistance phenotypes was carried out by the Kirby-Bauer methods [44]. MIC of methicillin was determined by E-test kits (AB Biodisk, Solna, Sweden). The results were categorized according to CLSI standards. Reference strains used as controls were *S. aureus *(ATCC 33591), *S. aureus *(ATCC 25923), and *S. epidermidis *(ATCC 12228) (Table 1).

### Primer design for pentaplex PCR assay

The 16S rRNA of *Staphylococcus *genus, and gene sequences for *fem*A, *mec*A and *luk*S of *S. aureus *were obtained from GenBank [45], for DNA sequence alignment and primer design. The ClustalW program in Vector NTI version 9.0 software (Invitrogen, Carlsbad, CA, USA) was used to align the DNA sequences. The conserved and non-conserved regions of the DNA sequence alignments were visualized using GeneDoc software [46].

Based on the conserved regions of the alignment, specific primer pairs were designed to amplify the *Staphylococcus *genus. Specific primers of *S. aureus *species were designed based on the non-conserved regions of *fem*A gene sequences. Methicillin-resistance specific primers were designed based on the conserved regions of *mec*A DNA sequences. For the PVL-encoding gene, specific primers were designed based on *luk*S gene. The five primer pairs (Research Biolabs, KL, Malaysia) were designed in such a way that the PCR products ranged from 151 to 759 bp. The specificity of the designed primers was checked using BLAST, which is available at the GenBank website [47]. The primer sequences for the five genes and expected PCR product sizes are shown in Table 2. A primer pair based on *hem*M gene was designed (759 bp) and was used as an internal control (Table 2).

**Table 2:** Sequences of primers used for the pentaplex PCR.

Gene	Primer Name	5'---------------------------------3'	Gen Bank accession number	Product size
**Internal**	IC-F	AGCAGCGTCCATTGTGAGA	AF227752	759 bp
**control *hem*M**	IC-R	ATTCTCAGATATGTGTGG		

**16S rRNA**	16S rRNA-F	GCAAGCGTTATCCGGATTT	D83356	597 bp
	16S rRNA-R	CTTAATGATGGCAACTAAGC		

***fem*A**	*fem*A-F	CGATCCATATTTACCATATCA	CP000255	450 bp
	*fem*A-R	ATCACGCTCTTCGTTTAGTT		

***mec*A**	*mec*A-F	ACGAGTAGATGCTCAATATAA	NC_003923M	293 bp
	*mec*A-R	CTTAGTTCTTTAGCGATTGC		

***luk*S**	*lukS*-F	CAGGAGGTAATGGTTCATTT	AB186917	151 bp
	*lukS*-R	ATGTCCAGACATTTTACCTAA		

### Pentaplex PCR assay

DNA-contamination is a major problem encountered in the routine use of the PCR; we followed all contamination prevention measures in the PCR daily work to avoid pre and post-PCR contamination [48].

The monoplex PCR for each gene and the pentaplex PCR assay were standardized using genomic DNA extracted from reference *Staphylococcus *spp. A mixture of DNAs from two reference strains, namely *S. aureus *(ATCC 33591) and *S. aureus *(ATCC 25923), which contained the four genes of interest was used as a positive control. DNase-free distilled water was used as a negative control. In addition, a plasmid pCR^® ^2.1-TOPO (Invitrogen) that contained *hem*M gene (1 pg) was used as a template for the internal control. To rule out false-negative results, an internal control (primer pair and template) was incorporated in every reaction mixture including negative controls.

Diagnostic evaluation of the pentaplex PCR was done using the lysates from 230 clinical isolates. The isolated colonies from blood agar were inoculated into LB broth and incubated at 37°C for 24 h. Bacterial lysates for PCR were prepared by centrifuging the 100 μl culture at 10,000 × *g *for 3 min; the supernatant was removed and the pellets were resuspended in 100 μl DNase-free distilled water. The suspensions were boiled in a water bath for 10 min and centrifuged again at 10,000 × *g *for 3 min. Then, 2 μl of the supernatants (lysates) was used in the pentaplex PCR assays.

The optimized concentration of primer for each gene (0.6 pmol 16 S rRNA, 0.8 pmol *fem*A S. *aureus*, 1.0 pmol *mec*A, 0.6 pmol *luk*S, and 0.8 pmol *hem*M) was used in the pentaplex PCR. The other components used in the PCR were 200 μM dNTPs, 3.13 mM MgCl_2_, 1× PCR buffer and 0.75 U *Taq *DNA polymerase (Fermentas, Vilnius, Lithuania). The PCR was carried out using a Mastercycler Gradient (Eppendorf, Hamburg, Germany) with one cycle of initial denaturation at 94°C for 3 min, 30 cycles of denaturation at 94°C for 30 s, annealing for 30 s at 60°C, and extension at 72°C for 30 s, followed by an extra cycle of annealing at 60°C for 30 s, and a final extension at 72°C for 5 min. The PCR products were analyzed by electrophoresis on 1.5% low EEO agarose gels (Promega, Madison, WI, USA), with ethidium bromide at 100 V for 75 min. PCR products were visualized under UV illumination and photographed using an image analyzer (ChemiImager 5500; Alpha Innotech, San Leandro, CA, USA).

### Evaluation of pentaplex PCR assay

Analytical specificity was evaluated using DNA lysates prepared from pure cultures of 10 phenotypically and genotypically well-characterized *Staphylococcus *spp. and 10 non-staphylococcal Gram-positive and 13 Gram-negative strains obtained from different sources (Table 1). The analytical sensitivity was evaluated using various concentrations of genomic DNA starting from 1 μg to 10 pg and lysate starting from 10^8 ^to 10^3 ^CFU/ml obtained from a reference strain, *S. aureus *(ATCC 33591). The diagnostic evaluation of the pentaplex PCR was carried out using 230 clinical isolates. The results were compared with the conventional microbiological, biochemical, and antimicrobial susceptibility E-test which were considered as the gold standard [20].

### Statistical analysis

The clinical sensitivity, specificity, and positive and negative predictive values of the pentaplex PCR were calculated based on the CLSI Guidelines for Molecular Diagnostic Methods for Infectious Diseases [20].

## Authors' contributions

HALT carried out the DNA sequence alignment, designed the primers, developed the multiplex PCR, analyzed clinical samples and drafted the manuscript. CYY contributed to the multiplex PCR optimization. AALK contributed to the primer design and data analysis. HH was involved in the initial study design in protocol development and selection of genes. KKBS contributed to the manuscript revision. KALJ participated in the study design and critically edited and revised the manuscript. MR conceived and coordinated the study, helped in DNA sequence analysis, primer design and data analysis, and drafted the manuscript. All authors read and approved the final manuscript.
